# Genes Related to Motility in an Ionizing Radiation and Estrogen Breast Cancer Model

**DOI:** 10.3390/biology13110849

**Published:** 2024-10-22

**Authors:** Tania Koning, Gloria M. Calaf

**Affiliations:** Instituto de Alta Investigación, Universidad de Tarapacá, Arica 1000000, Chile; tkoning@academicos.uta.cl

**Keywords:** cell motility, breast cancer, radiation, estrogen receptors, a disintegrin and metalloprotease (ADAM12), cysteine-rich angiogenic inducer 61 (CYR61), fibronectin leucine-rich transmembrane protein 2 (FLRT2), slit homolog 2 protein (SLIT2), vascular non-inflammatory molecule-1 (VNN1), myosin light chain kinase (MYLK), microtubule-associated protein 1B (MAP1B) and tubulin alpha-1A (TUBA1A)

## Abstract

Breast cancer is a major global health concern as it is the primary cause of cancer death for women. Environmental radiation exposure and endogenous factors such as hormones increase breast cancer risk, and its development and spread depend on cell motility and migration. The expression of genes associated with cell motility, such as *ADAM12*, *CYR61*, *FLRT2*, *SLIT2*, *VNN1*, *MYLK*, *MAP1B*, and *TUBA1A*, was analyzed in an experimental breast cancer model induced by radiation and estrogen. The results showed that *TUBA1A*, *SLIT2*, *MAP1B*, *MYLK*, and *ADAM12* gene expression increased in the irradiated Alpha3 cell line but not in the control or the malignant Tumor2 cell line. Bioinformatic analysis indicated that *FLERT2*, *SLIT2*, *VNN1*, *MAP1B*, *MYLK*, and *TUBA1A* gene expressions were found to be higher in normal tissue than in tumor tissue of breast cancer patients. However, *ADAM12* and *CYR61* expressions were found to be higher in tumors than in normal tissues, and they had a negative correlation with *ESR1* gene expression. Concerning *ESR2* gene expression, there was a negative correlation with *CYR61*, but there was a positive correlation with *FLRT2*, *MYLK*, *MAP1B*, and *VNN1*. Finally, a decreased survival rate was observed in patients exhibiting high expression levels of *TUBA1A* and *MAP1B.* These genes also showed a negative ER status, an important parameter for endocrine therapy. The genes related to motility were affected by ionizing radiation, confirming its role in the initiation process of breast carcinogenesis. In conclusion, the relationship between the patient’s expression of hormone receptors and genes associated with cell motility presents a novel prospect for exploring therapeutic strategies.

## Simple Summary

Breast cancer is the leading cause of cancer death in women, with risk factors including environmental radiation exposure and hormones. This study examined the expression of genes associated with cell motility necessary for cancer progression in cells of varying malignancy exposed to radiation and estrogen. The expression of these genes was also compared with patient data provided by other researchers. The results indicated that these genes showed increased expression in response to radiation alone or in combination with estrogen. Additionally, in patients, the expression of most of these genes was found to be higher in normal tissues adjacent to the tumor ones. The estrogen receptor alpha (*ESR1*) and the estrogen receptor beta (*ESR2*) genes, which are significant for patient characterization and treatment, were used to carry out correlations. The analysis of the patient data revealed a low correlation between the genes related to motility and *ESR1* expression, whereas a positive correlation was observed between the genes in this study and *ESR2* expression. Furthermore, patients with high expression levels of the microtubule-associated protein 1B gene or tubulin alpha-1A gene exhibited a decreased survival rate. These findings suggest that there may be potential for exploring new therapeutic strategies based on the association between patients’ expression of hormone receptors and genes associated with cell motility.

## 1. Introduction

Female breast cancer was the second leading cause of global cancer incidence in 2022, with an estimated 2.3 million new cases [1]. Among women, breast cancer is the most diagnosed cancer, and it is the leading cause of cancer deaths worldwide (6.9% of all cancer deaths), in 157 countries in terms of incidence, and in 112 countries in terms of mortality [1]. The risk of breast cancer is increased by endogenous variables, like hormones, and exogenous factors, like exposure to radiation or chemicals in the environment [2]. The hormonal estrogen receptor (ER) is the most important biomarker in breast oncology, and approximately 75% of breast cancers are clinically diagnosed as estrogen receptor positive (ER+) [3]. The ER status plays a key role in clinical decision-making and outcome prediction for patients with breast cancer. This is due to its crucial role in determining if a patient is likely to have a positive response to endocrine therapy [4]. The degree of hormone receptor positivity is connected with both the endocrine sensitivity and the efficacy of hormonal treatment [5].

The nuclear ER is encoded by the estrogen receptor alpha gene (*ESR1*) and the estrogen receptor beta gene (*ESR2*), whose corresponding protein products are ERα and ERβ, respectively [6]. The progression of breast cancer is positively associated with increased ERα activity [7]. On the other hand, the similarity between the ligand binding domain and DNA binding domain of ERβ supports its carcinogenic activity (96% and 60%, respectively) with that of ERα, suggesting that their roles may be comparable but not identical [8]. ERβ is usually abundant in normal breast epithelial cells [9]; however, the positive rate of ERβ in breast cancer has been reported to be over 60% [10,11].

Ionizing radiation (IR) is another risk factor in the development of breast cancer. Numerous epidemiological and experimental studies have shown the high sensitivity of the mammary gland to this factor [12]. IR consists of particles and photons that possess sufficient energy to ionize human body atoms, causing chemical changes crucial to cellular function. For example, IR can modify and destroy DNA, RNA, and components of the cell membrane, such as lipids and proteins, either via direct ionization or through the process of water radiolysis [13]. The importance of IR is so profound that it has been used as an initiator and promoter in cell models of human and animal cell lines [14,15].

A critical event in the progression of cancer leading to metastasis is the acquisition of a motile phenotype by the cell [16]. Therefore, the genes selected in this study are associated with cell motility. Cell motility is a strategy that triggers genes and protein activation to produce movement in cells in response to stimuli. This involves the reorganization of the cytoskeleton through the activation of receptors and intracellular signals, the secretion and activation of enzymes that degrade the cellular matrix, and, finally, the adhesion of one end of the cell in a forward direction and detachment of the back end.

Among others, a disintegrin and metalloprotease (ADAM) is a family of integral membrane or secreted glycoproteins that play important roles in regulating cell adhesion and migration [17,18]. ADAMs are responsible for the proteolytic processing of a variety of cell surface receptors and signaling molecules, including notch receptors and their ligands, ligands of the epidermal growth factor receptor (EGFR), and tumor necrosis factor and its respective receptors [17,18].

The cysteine-rich angiogenic inducer 61 (CYR61) is a secreted protein 1 that belongs to the cysteine-rich 61/connective tissue growth factor/nephroblastoma (CCN) gene family of survival and angiogenic regulators [19,20]. Whereas all CCN proteins have been shown to mediate functions such as cell proliferation, migration, adhesion, differentiation, and extracellular matrix formation, CYR61 has a unique ability to regulate more complex processes, such as angiogenesis and tumorigenesis [21,22,23,24,25,26,27,28,29].

Another membrane protein selected in this study is FLRT2 (fibronectin leucine-rich transmembrane protein 2), a protein that interacts with several other proteins, such as ROBO117, LPHN3, and UNC [30,31]. FLRT2 has been implicated in differentially regulated processes between males and females, such as prostate cancer and several primarily female and hormone-related physiological and pathophysiological processes in humans, including menarche [32] and breast cancer [33,34].

On the other hand, the extracellular-matrix-secreted glycoprotein SLIT2 has been linked to neuronal development, myogenesis, leukocyte chemotaxis, and cancer [35,36]. Initially identified as an axon guidance cue, SLIT2 has also been shown to regulate mammary gland growth and development [37].

The vascular non-inflammatory molecule-1 (vanin-1) has also been included in this study. Vanin-1 is an epithelial glycosylphosphatidylinositol (GPI)-anchored ectoenzyme that catalyzes the cleavage of pantetheine into the aminothiol cysteamine and pantothenic acid (vitamin B5, the coenzyme A precursor) [38,39].

Additionally, proteins related to the reorganization of the cellular cytoskeleton have also been considered, such as the myosin light chain kinase (MYLK), which is a protein kinase, whose main published function is to phosphorylate and, thereby, activate myosin light chain (MLC2). MCL2 plays a significant role in cell morphology, contraction, motility, and other membrane events like apoptotic blebbing [40]. *MYLK* expression was downregulated in breast cancer, and loss of *MYLK* led to the disruption of cell–cell adhesion and invasive behavior of breast epithelial cells [41].

Microtubules are crucial components that play a role in numerous cell activities, such as preserving cell shape, vesicle transport, motility control, and cell division. The tubulin alpha-1A chain (TUBA1A) undergoes polymerization to form microtubules, essential during mitosis in cellular division. The swift multiplication of cancer cells relies heavily on the dynamic cell process of tubulin polymerization/depolymerization [42], which is why it has been a pharmacological target to stop cancer progression. Microtubule-associated protein 1, MAP1B, is a protein that regulates microtubule dynamics, binding, and stabilization [43,44]. MAP1A and MAP1B are expressed predominantly in the nervous system [45,46]. The rapid proliferation of cancer cells is highly dependent on the dynamic cellular process of tubulin polymerization/depolymerization; therefore, this protein plays a vital role in this process.

This work aimed to study and compare the gene expression associated with cell motility from an experimental breast cancer model induced by radiation and estrogen, including a disintegrin and metallopeptidase 12 gene (*ADAM12*), the cysteine-rich angiogenic inducer 61 gene (*CYR61*), the fibronectin leucine rich transmembrane protein 2 gene (*FLRT2*), the slit guidance ligand 2 gene (*SLIT2*), the vanin-1 gene (*VNN1*), the myosin light chain kinase gene (*MYLK*), the microtubule-associated protein 1B gene (*MAP1B*), and the tubulin alpha 1a gene (*TUBA1A*). This study then connected these findings with clinical data procured from online breast cancer databases.

## 2. Materials and Methods

### 2.1. An Experimental Radiation and Estrogen-Induced Breast Cancer Model

The MCF-10F cells were acquired from ATTC, and the cell lines were developed at the Radiological Research Accelerator Facility of Columbia University by the authors; the information can be found in Calaf and Hei, 2000 [14]. The MCF-10F cells that were grown exponentially were plated in 60 mm diameter stainless steel rings with a 6 μm mylar bottom, three days before irradiation, at a density of 3 × 10^5^ cells. As reported in [47], graded dosages of accelerated ^4^He ions at 150 KeV/μm were applied to the cells using the 4 Me V van de Graaff accelerator at the Columbia University Radiological Research Facilities. These high-energy particles are similar in LET value to the α particles released by the progeny of radon. The MCF-10F cells were cultured for ten to fifteen passages, with an interval of twelve to fourteen weeks, before being exposed to a single or double dose of 30, 60, or 100 cGy of 4He ions. The irradiated cultures were frozen as future stock, while they were subcultured to ascertain growth kinetics and expanded in culture to test for altered phenotypes. Then, the residual cells underwent repeated passages for radiation therapy after being sampled for diverse transformed phenotypes. After that, the cells were cultivated with or without estrogen [14].

The MCF-10F cells were exposed to either a single 60 cGy dose or 60/60 cGy doses of alpha particles, and they were examined at various phases of transformation [14]. The cell lines used in this model were the following: the parental cell line MCF-10F (Ct); an estrogen cell line (E), which was MCF-l0F continuously grown with 17β-estradiol (Sigma Chemical Co., St. Louis, MO, USA) at 10^−8^ M; a malignant and non-tumorigenic cell line (60/60 cGy) named Alpha3 (A3); a tumorigenic cell line (60/60 cGy plus estrogen) named Alpha5 (A5); and the Tumor2 cell line (T2), which was generated by injecting an Alpha5 cell line xenograft into nude mice. These cell lines were cultivated for up to 10 months with or without estrogen. The animals injected with the cell lines E and Alpha3 did not form mammary tumors; however, A5 and Tumor 2 were tumorigenic in the model and SCID animal [14]. The phenotypical characteristics of an experimental breast cancer model were previously summarized [48].

### 2.2. Differential Expression of Genes Associated with Motility in a Breast Cancer Model

Using an Affymetrix (U133A) oligonucleotide microarray (Affymetrix, Santa Clara, CA, USA), differentially expressed genes were measured in the cell lines to identify the gene expression of the following genes related to cell motility: a disintegrin and metallopeptidase 12 gene (*ADAM12*), the cysteine-rich angiogenic inducer 61 gene (*CYR61*), the fibronectin leucine rich transmembrane protein 2 gene (*FLRT2*), the slit guidance ligand 2 gene (*SLIT2*), the vanin-1 gene (*VNN1*), the myosin light chain kinase gene (*MYLK*), the microtubule-associated protein 1B gene (*MAP1B*), and the tubulin alpha 1a gene (*TUBA1A*). Such an Affymetrix array rendered 14,500 genes [49] using the cell lines provided by the model mentioned above. Those cell lines were analyzed in pairs according to the following criteria: (i) to compare the effect of estrogen alone versus the control in MCF-10F/E to analyze the estrogen effect; (ii) to compare the effect of the ionizing radiation alone versus the control in MCF-10F/Alpha3 to analyze radiation alone; (iii) to compare the estrogen when ionizing radiation was used in E/Alpha5 to assess the radiation effect; (iv) to compare the ionizing radiation alone with the environment in the athymic animal: Alpha 3/Tumor2 to assess the role of the microenvironment and radiation alone; and (v) to compare both estrogen and ionizing radiation with the environment in the athymic animal: Alpha5/Tumor2.

The gene expression in the arrays was quantitatively assessed using the Affymetrix GeneChip Operating Software (GCOS) v1.0 ST. The analysis was performed using the SPLASH (structural pattern localization analysis by sequential histograms) discovery approach with a false discovery rate of 0.05 [50]. The Affymetrix U133A oligonucleotide microarray experiment was conducted once and contained 14,500 genes.

### 2.3. Differential Expression of Genes Associated with Motility Between Different Breast Tumor Types and Normal Tissues

The TIMER2.0 webpage (http://timer.cistrome.org/) [51] was used for estimating differential gene expression between tumors and adjacent normal tissues. *ADAM12*, *CYR61*, *FLRT2*, *SLIT2*, *VNN1*, *MYLK*, *MAP1B*, and *TUBA1A* gene expression levels across The Cancer Genome Atlas (TCGA) breast tumor are displayed using box plots. The statistical significance computed by the Wilcoxon test is annotated by the number of stars (*: *p* < 0.05; **: *p* < 0.01; ***: *p* < 0.001).

### 2.4. Protein Expression Associated with Cell Motility in Breast Tissues

UALCAN (https://ualcan.path.uab.edu/index.html) provides protein expression analysis options using data from the Clinical Proteomic Tumor Analysis Consortium (CPTAC) and the International Cancer Proteogenomic Consortium (ICPC) datasets. To analyze protein expression levels, the University of Alabama at Birmingham Cancer Data Analysis Portal (UALCAN, http://ualcan.path.uab.edu/ accessed on 5 June 2024) was used [52,53]. This platform uses Clinical Proteomic Tumor Analysis Consortium (CPTAC) data. Specifically, the proteomics module was chosen to enter the genes of interest, and the breast cancer dataset total protein was filtered. The result was delivered in a graphical form where the Z-values represent standard deviations from the median across samples for breast cancer. Log2 Spectral count ratio values from CPTAC were first normalized within each sample profile and then normalized across samples.

### 2.5. Correlation Between ESR1 and ESR2 Genes and Motility Gene Expression

The TIMER2.0 webpage for The Cancer Genome Atlas (TCGA) (http://timer.cistrome.org/) was used. Briefly, the tool explores the correlation between a gene of interest (*ESR1* or *ESR2*) with a list of genes (motility genes) in various cancer types. Hence, the information provided was filtered to study breast cancer (BRCA) subtypes, such as Basal, Her2, Lum-A, and Lum-B, and to identify the relationship between *ESR1* or *ESR2* and motility genes (*ADAM12*, *CYR61*, *FLRT2*, *SLIT2*, *VNN1*, *MYLK*, *MAP1B*, and *TUBA1A*). The value corresponds to the purity-adjusted Spearman’s rho in breast cancer. The red color indicates a statistically significant positive correlation (Spearman’s, *p* < 0.05), blue indicates a statistically significant negative correlation (Spearman’s, *p* < 0.05), and gray denotes a nonsignificant result.

### 2.6. Gene Expression Related to Motility and Estrogen Receptor Status in TCGA Breast Cancer

The University of California, Santa Cruz (http://xena.ucsc.edu/) UCSC Xena Functional Genomics Explorer allows for us to examine functional genomic datasets for correlations between genomic and/or phenotypic [54]. For the explorative study, TCGA Breast Cancer (BRCA) Genomic data were considered, and *ADAM12*, *CYR61*, *FLRT2*, *SLIT2*, *VNN1*, *MYLK*, *MAP1B*, and *TUBA1A* genes were chosen in the webpage. Next, gene expression was selected as the first variable, and Phenotype ER_status_ nature2012 was selected as the second variable. Then, comparative subgroups analysis gives a box plot with statistical significance computed by the one-way ANOVA test; *p* < 0.05 was considered significant.

### 2.7. Relationship Between Genes Associated with Motility and Clinical Aspects

The gene outcome provided by the TIMER2.0 webpage allows for exploring associations between gene expression and tumor features in The Cancer Genome Atlas (TCGA) to understand the clinical relevance of gene expression across BRCA subtypes. This exploration uses the Cox proportional hazard model to evaluate the outcome significance of gene expression. The heatmap shows the normalized coefficient (Z-score) of the gene in the Cox model. The red color indicates a statistically significant increased risk (Z-score, *p* < 0.05), and gray denotes a nonsignificant result. The pop-up by the red Z-score of the heatmap table shows the Kaplan–Meier curve of the gene, with one curve representing patients with high gene expression and another representing patients with low expression. The Kaplan-Meier curve is given by cumulative survival versus time to follow-up (months). The data from Human Protein Atlas (HPA) (https://www.proteinatlas.org/ accessed on 12 June 2024) were used to gain insights into the immunohistochemistry of MAP1B and TUBA1A in both malignant and normal tissues. Four images were chosen from each of the study groups (automatically given by the HPA), and the expression of these proteins in the tissues was graphed using the IHC Toolbox plugin of ImageJ software version 1.53t.

## 3. Results

The microarray data from these experimental model cells served as the basis for selecting the genes for this study. The gene expression was compared with online databases containing patient gene expression information. These analyses encompassed both gene and protein expression in normal as well as tumor breast tissues. Additionally, the expression of genes associated with cell motility was correlated with those encoding estrogen receptors. These findings were then compared with the phenotypic estrogen receptor status (ER status) parameter. Finally, this study assessed whether the expression of these genes influenced the prognosis of breast cancer patients.

### 3.1. Differential Expression of Genes Related to Cell Motility in an Experimental Radiation and Estrogen-Induced Breast Cancer Model

The cell lines used in this study from the experimental breast cancer model correspond to the control MCF-10F cell line (Ct); the estrogen cell line (E), MCF-l0F continuously grown with 17β-estradiol; the Alpha3 (A3), an irradiated, malignant, and non-tumorigenic cell line; the Alpha5 (A5), an irradiated and tumorigenic cell line cultured in the presence of 17β-estradiol; and the Tumor2 (T2) cell line, which was generated by injecting an Alpha5 cell line xenograft into nude mice. Then, differential gene expression was evaluated in the following pairs: MCF-10F/Estrogen (Ct/E), Control/Alpha3 (Ct/A3), Estrogen/Alpha5 (E/A5), Alpha3/Alpha5 (A3/A5), Alpha5/Tumor2 (A5/T2), and Alpha3/Tumor2 (A3/T2).

**Figure 1:**
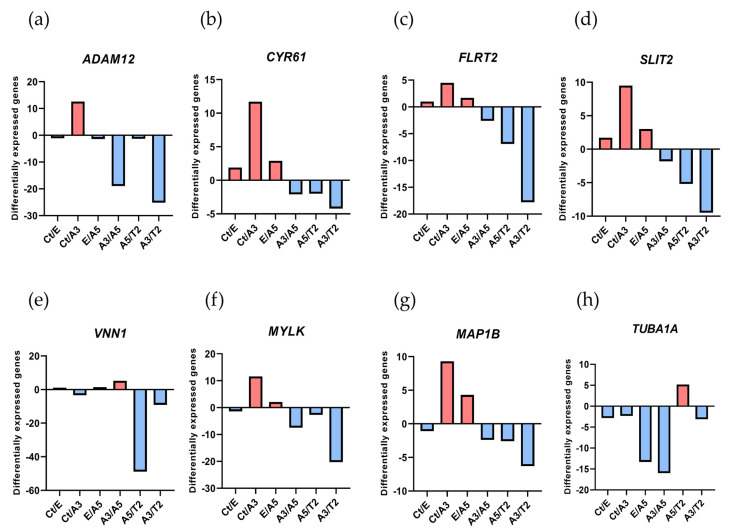
Relative expression of cell motility genes from Affymetrix array (U133A) in an experimental breast cancer model induced by radiation and estrogen. The analyzed genes were (**a**) *ADAM12*, (**b**) *CYR61*, (**c**) *FLRT2*, (**d**) *SLIT2*, (**e**) *VNN1*, (**f**) *MYLK*, (**g**) *MAP1B*, and (**h**) *TUBA1A* in MCF-10F/Estrogen (Ct/E); Control/Alpha3 (Ct/A3); Estrogen/Alpha5 (E/A5); Alpha3/Alpha5 (A3/A5); Alpha5/Tumor2 (A5/T2); and Alpha3/Tumor2 (A3/T2) cell lines. Red indicates a positive value and blue a negative one. The graphs were obtained from a cluster-dendrogram repository of gene expression from our laboratory for this article.

The Affymetrix array (Figure 1) showed that the gene expressions were predominantly higher in the A3 cell line than in the Ct, A5, and T2 cell lines.

The A5 cell line showed more expression of *FLRT2* and *MAP1B* in comparison with the E and T2 cell lines, and *VNN1* expression was higher in A5 than in T2, and such expression increased in the A3 cell line compared to the T2 cell line as well. The A3 cell line exhibited a significant radiation effect, especially in *SLIT2* expression, when compared to the malignant and tumorigenic A5 and T2 cell lines. The E and A3 cell lines had a higher *TUBA1A* gene expression than the A5 cell line.

### 3.2. Expression of Genes Related to Cell Motility in Tumor and Normal Breast Tissues According to Patient Database

**Figure 2:**
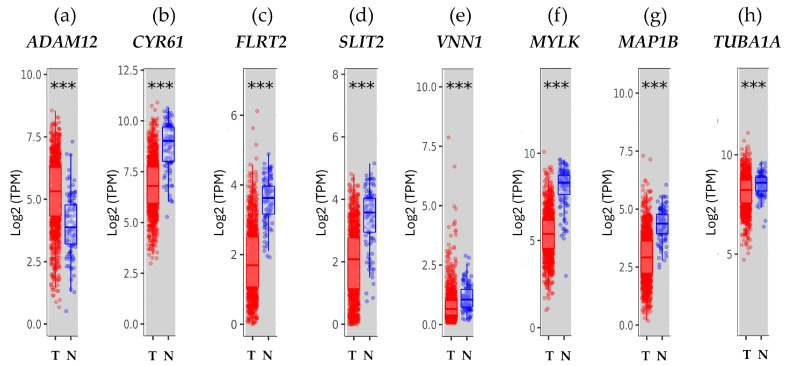
Gene expression related to cell motility in tumor and normal tissues in breast cancer patients. The box plots show the distribution of gene expression levels quantified in transcripts per million (TPM) of (**a**) *ADAM12*, (**b**) *CYR61*, (**c**) *FLRT2*, (**d**) *SLIT2*, (**e**) *VNN1*, (**f**) *MYLK*, (**g**) *MAP1B*, and (**h**) *TUBA1A* in tumors versus normal tissues (Wilcoxon test,***: *p* < 0.001) estimated by TIMER2.0 in breast invasive carcinoma tumor (T) (*n* = 1093) and adjacent normal tissue (N) (*n* = 112).

Figure 1 shows the gene expression levels associated with cell motility in cell lines derived from an experimental breast cancer model. To determine the contribution of these genes in breast cancer, their expression was compared in tumor and normal tissues using the TIMER2.0 online database to analyze (Figure 2).

The results, as shown in Figure 2, indicated that *ADAM12* expression was higher in the tumor than in the adjacent normal tissue (Figure 2a). However, *CYR61*, *FLRT2*, *SLIT2*, *VNN1*, *MAP1B*, *MYLK*, and *TUBA1A* gene expressions were high in normal adjacent tissue in comparison with those in breast tumor tissue (Figure 2a–h). This statistically significant analysis was calculated using the Wilcoxon test, *p* < 0.001.

### 3.3. Protein Expression Related to Cell Motility in Normal and Primary Tumor Samples

**Figure 3:**
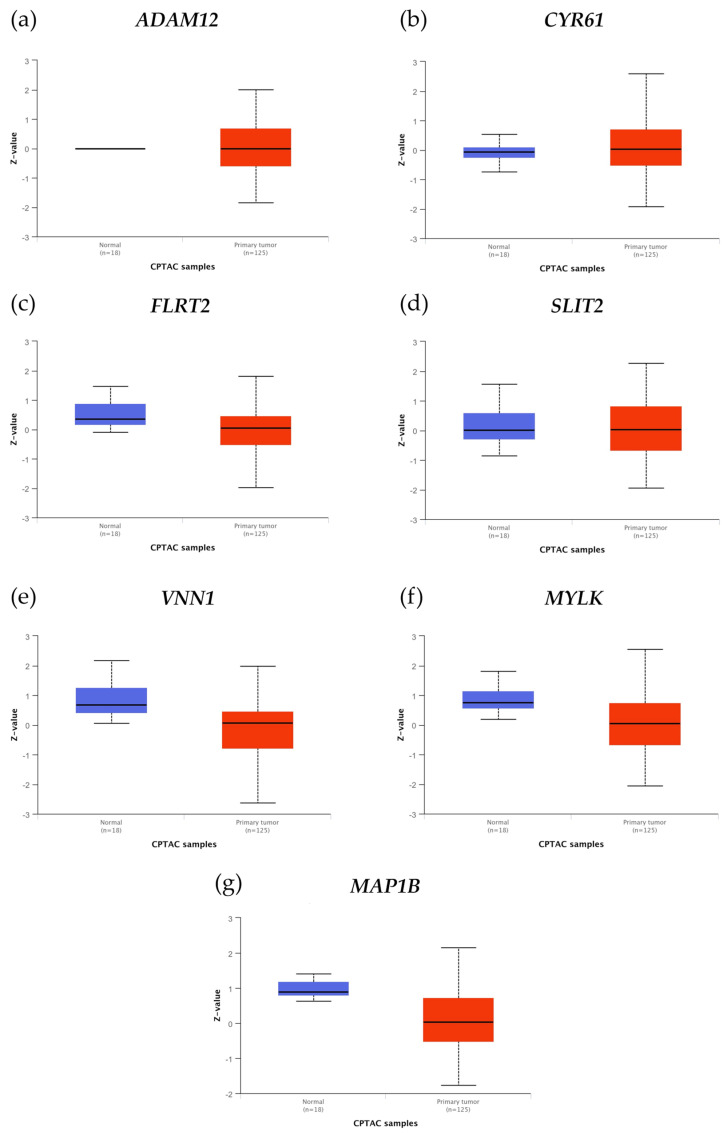
Protein expression in normal and primary tumor samples. The box plots show the distribution of protein expression levels of (**a**) ADAM12, (**b**) CYR61, (**c**) FLRT2, (**d**) SLIT2, (**e**) VNN1, (**f**) MYLK, and (**g**) MAP1B. Z-values represent standard deviations from the median across samples for the given cancer type. Log2 Spectral count ratio values from CPTAC were first normalized within each sample profile and then normalized across samples. Normal samples (*n* = 18) and primary tumors (*n* = 125).

The protein expression of genes related to cell motility was evaluated using data sourced from the Clinical Proteomic Tumor Analysis Consortium (CPTAC) as shown in Table 1 and Figure 3.

The results showed that ADAM12, FLRT2, SLIT2, VNN1, MYLK, and MAP1B protein expression levels were higher in normal samples (*n* = 18) than in primary tumors (*n* = 125). However, CYR61 expression level was higher in primary tumors than in normal samples. TUBA1A was not identified by CPTAC as part of the breast dataset.

### 3.4. Correlation Between ESR1, ESR2, and Genes Associated with Cell Motility in Breast Cancer Subtypes

**Figure 4:**
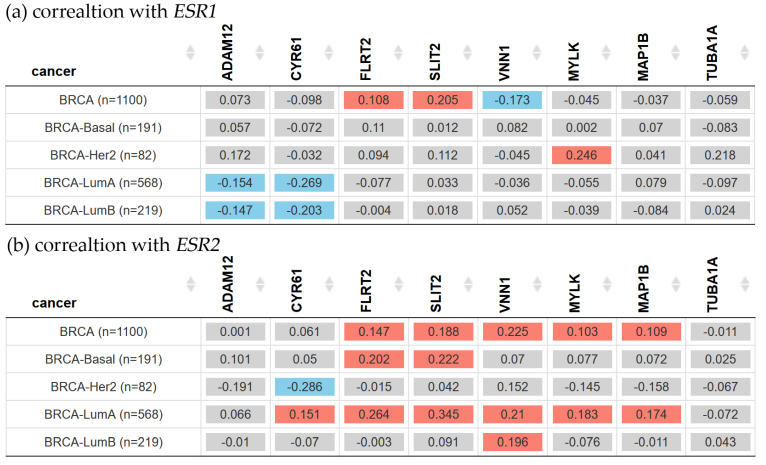
Correlation between *ESR1—ESR2* and cell motility genes in breast cancer types. Correlation was performed using TIMER2.0 in subtypes of breast carcinoma patients. (**a**) Correlation of *ESR1* with *ADAM12*, *CYR61*, *FLRT2*, *SLIT2*, *VNN1*, *MYLK*, *MAP1B*, and *TUBA1A* in breast invasive carcinoma subtypes. (**b**) Correlation of *ESR2* with the same genes. Heatmap gives the purity-adjusted partial Spearman’s rho value as the degree of their correlation. The red color indicates a statistically significant positive correlation (Spearman’s, *p* < 0.05), blue indicates a statistically significant negative correlation (Spearman’s, *p* < 0.05), and gray denotes a nonsignificant result.

The estrogen receptor (ER) is a key hormonal biomarker in breast cancer studies. In this sense, the correlation between the genes associated with cell movement and the genes responsible for encoding ERα (*ESR1*) and ERβ (*ESR2*) was evaluated (Figure 4).

Figure 4a shows that both *ADAM12* and *CYR61* gene expressions had a negative correlation with *ESR1* in Luminal A and Luminal B patients. However, the *MYLK* gene expression had a positive correlation with *ESR1* gene expression in Her2 patients. According to Figure 4b, *FLRT2* and *SLIT2* gene expression had a positive correlation with *ESR2* in Basal and Luminal A patients. *MYLK* and *MAP1B* gene expression levels had a positive correlation with *ESR2* in Luminal A patients. *VNN1* gene expression had a positive correlation with *ESR2* in Luminal A and Luminal B patients, whereas gene expressions of *CYR61* and *ESR2* were negatively correlated in Her2 but positively correlated in Luminal A patients.

### 3.5. Expression of Genes Associated with Cell Motility According to Estrogen Receptor Status in TCGA Breast Cancer Patients

**Figure 5:**
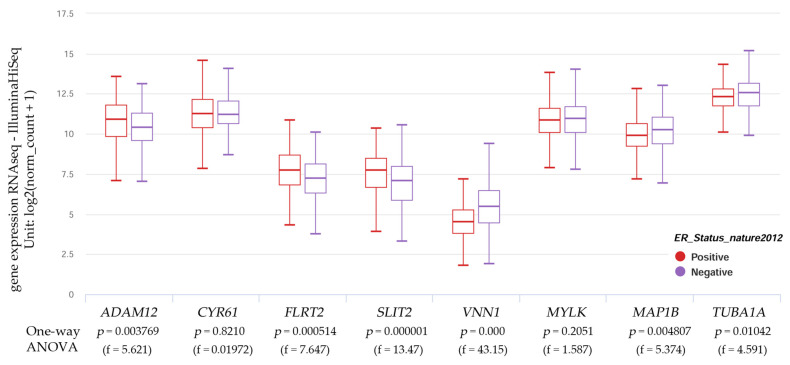
Genes associated with cell motility and estrogen receptor status in TCGA breast cancer. Xena graph shows a box plot corresponding to the gene expressions of *ADAM12*, *CYR61*, *FLRT2*, *SLIT2*, *VNN1*, *MYLK*, *MAP1B*, and *TUBA1A* in breast cancer (cohort: TCGA Breast Cancer (BRCA), *n* = 782) phenotypically classified by nature 2012 for estrogen receptor status (one-way ANOVA, *p* < 0.05). The red box plot corresponds to estrogen receptor-positive breast cancers, and the purple box plot corresponds to estrogen receptor-negative breast cancer. Raw data were extracted from the University of California, Santa Cruz, UCSC Xena Functional Genomics Explorer (https://xena.ucsc.edu/), accessed on 8 April 2023.

UCSC Xena is a web tool that enables the association of genomic and phenotypic data. Figure 5 examines the correlation between the expression of genes linked to cell movement (genomic data) and the ER status (phenotypic data) in individuals diagnosed with breast cancer.

The results indicated that the gene expression of *ADAM12*, *FLRT2*, and *SLIT2* was higher in ER alpha-positive cancers. In contrast, *MAP1B*, *TUBA1A*, and *VNN1* gene expression levels were higher in patients who did not express the estrogen receptor (ER negative). On the other hand, the expression of the *CYR61* and *MYLK* genes did not have significant variations between patients with estrogen receptor-positive and -negative breast cancer.

### 3.6. Cell Motility Gene and Protein Expression in Breast Cancer Subtypes and Clinical Relevance

**Figure 6:**
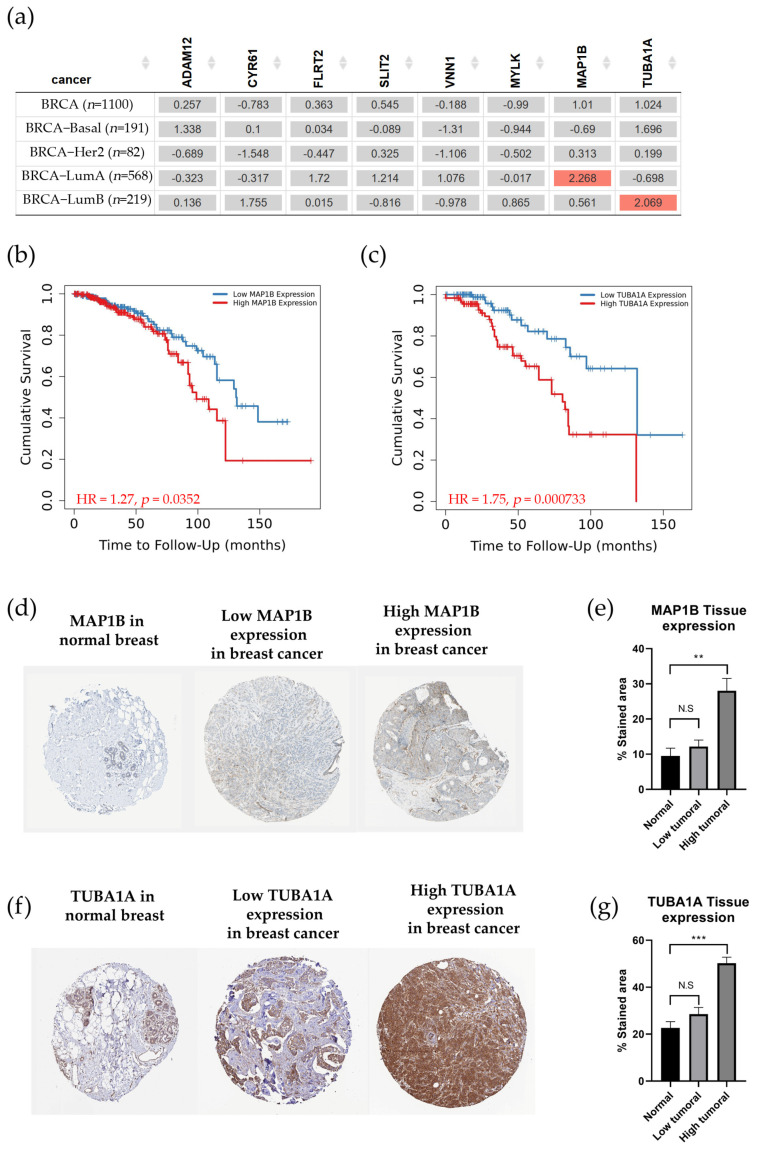
Clinical relevance of genes related to cell motility across breast cancer subtypes. (**a**) The heatmap table shows the Z-score from the TIMER2.0 Gene Outcome module that analyzed genes such as *ADAM12*, *CYR61*, *FLRT2*, *SLIT2*, *VNN1*, *MYLK*, *MAP1B*, and *TUBA1A* across breast cancer subtypes. The red color indicates a statistically significant increased risk (Z-score, *p* < 0.05), and gray denotes a nonsignificant result. The Kaplan–Meier (KM) curve gives cumulative survival versus time to follow-up (months) of (**b**) *MAP1B* and (**c**) *TUBA1A* genes. (**d**) Representative immunohistochemical of MAP1B tissue expression in normal breast, low expression, and high expression obtained from the Human Protein Atlas. (**e**) Quantification of MAP1B tissue expression, shown as a percentage of stained area (one-way ANOVA, **: *p* < 0.01). (**f**) Representative immunohistochemical of TUBA1A tissue expression in normal breast, low expression, and high expression obtained from the Human Protein Atlas. (**g**) Quantification of *TUBA1A* tissue expression, shown as a percentage of the stained area (one-way ANOVA, ***: *p* < 0.001).

The relevance of gene expression associated with cell motility in the survival of breast cancer patients was evaluated in Figure 6.

The results, as shown in Figure 6, indicated that *ADAM12*, *CYR61*, *FLTR2*, *SLIT2*, *VNN1*, and *MYLK* gene expressions did not show any significant values in the gene outcome module. However, the expression of the *MAP1B* and *TUBA1A* genes was positive in Luminal A and Luminal B patients, respectively. The results of the Kaplan–Meier curves showed that patients with high *MAP1B* expression had a decrease in the probability of survival by 80% at approximately 120 months (Figure 6b). On the other hand, patients with high *TUBA1A* expression did not survive within 120 months (Figure 6c). Additionally, protein expression was evaluated in the histological samples available in the Human Protein Atlas, where immunohistochemical samples of normal breast and tumors with low or high MAP1B and TUBA1A expression were compared in Figure 6d,f. The tissue expression of MAP1B and TUBA1A had no significant differences between normal breast samples and patients with low expression of those proteins. However, MAP1B expression showed an increase from 10 to 30% staining in the tissues (Figure 6e), while TUBA1A expression increased from 20 to 50% staining, as shown in Figure 6g.

## 4. Discussion

Cell motility and migration are critical features of invasive tumor cells and result from highly integrated multistep cellular events [55,56,57]. Migration is triggered by a promoting agent, leading to the formation of a forward protrusion that interacts with the extracellular matrix (ECM) to generate force and adhesion. This causes activation of cell-surface proteases at the rear of the leading edge, resulting in the cleavage of ECM components. Subsequently, the tension in the actomyosin cytoskeleton causes cellular contraction, breaking adhesion bonds at the trailing edge, thereby propelling cancer cells forward [58]. Hence, the findings of this study underline the importance of considering the experimental breast cancer model described herein, since few studies have addressed the impact of ionizing radiation on cellular motility in breast cancer.

The Affymetrix U133A oligonucleotide microarray indicated that *ADAM12* gene expression was higher in the A3 cell line than in the Ct, A5, and T2 cell lines, suggesting that ionizing radiation alone was involved in the transformation of the cell line, increasing the levels of *ADAM12*, according to the authors [59]. It is a disintegrin and metalloproteinase that contributes to the occurrence of many cancers. Supporting the importance of radiation in upregulating *ADAM12*, the authors demonstrated up-regulated *ADAM12* expression in normal and tumor skin of an animal experimental model after UV irradiation [60], and in CAL-27 and HN4 head and neck squamous cell carcinoma cells after being irradiated with X-rays [61].

On the other hand, the expression of the *ADAM12* gene was high in tumor tissue compared to normal tissue, which contrasted with the findings regarding protein expression. However, these findings are in line with a study that involved 127 Chinese patients and revealed that, in malignant breast cancer tissues, *ADAM12* mRNA levels were higher than in neighboring normal tissues [62]. Additionally, compared to control cells, human squamous cell carcinoma and cell lines from the same malignancies were shown to have higher levels of *ADAM12* RNA [63]. The fact that this protease is mostly secreted may account for its increased presence in normal tissues. *ADAM12* has also been found in the urine of breast cancer patients, with its protein levels being connected with cancer risk, stage, and disease status. The diverse physiologic functions that ADAMs carry out, such as promoting cell multiplication and cleaving cytokines, chemokines, or their receptors, explain their contribution to cancer progression, regardless of whether *ADAM12* levels are elevated in normal or tumor tissue [59].

The analysis of clinical data indicated that the correlation between the expressions of *ADAM12* and *ESR1* in patients with Luminal A and Luminal B breast cancer was negative; however, the expressions of *ADAM12* and *ESR2* were not statistically significant. However, ERα positive (phenotypic feature) patients showed higher expression of *ADAM12*, suggesting that *ADAM12* could be a marker for breast cancer if their ER status was positive. ADMA12 isoforms also gave a growth advantage to MCF-7 cells (ER+) even without estrogen stimulation. This implies that reducing ADAM12 levels alongside endocrine therapy could be a beneficial strategy in breast cancer treatment [64].

Authors have shown that CYR61 is a cell-associated as well as a secreted matricellular protein involved in tumor formation, growth, vascularization, angiogenesis, adhesion, drug resistance, migration, and invasion [65]. However, the role of radiation in CYR61 expression has been scarcely explored. In the present model, *CYR61* gene expression was higher in the irradiated A3 cell line than in the Ct and T2 cell lines, indicating the importance of ionizing radiation in breast carcinogenesis. A study showed that, in primary human fibroblast cell lines, *CYR61* gene expression decreased after exposure to 2 Gy γ-radiation, a type of IR [66]. On the other hand, UVB radiation (non-ionizing) stimulated the secretion of CYR61 in normal human dermal fibroblasts [67] and increased CYR61 mRNA and protein levels in human skin malignancies in vivo [68].

The analysis of clinical data indicated that *CYR61* gene expression was higher in normal breast tissue than in tumor breast tissue. The CYR61 expression was strongly correlated with markers for invasiveness and associated with the ability of breast cancer cells to invade in vitro and metastasize in vivo [24,25,26]. Such CYR61 overexpression and then progression to a metastatic phenotype have been signs of therapeutic resistance and mortality in breast cancer [69,70,71,72], but the precise mechanisms remain unknown. On the other hand, when correlating the expression of estrogen receptors with *CYR61*, the present study found that the *ESR1* expression levels were negatively correlated with *CYR61* in Luminal A and Luminal B patients. *ESR2* gene expression was also negatively correlated in Her2 breast cancer patients, whereas it was positively correlated in Luminal A patients. According to these findings, there was no correlation with the hormone receptor gene *ESR1*, which was further supported by the lack of statistically significant differences in breast cancer patients’ ER status. Such findings were corroborated by reports showing that *CYR61* was overexpressed in roughly 30% of patients with triple-negative breast cancer [24,25]. Nonetheless, the positive correlation shown in Luminal A patients between the *ESR2* gene and *CYR61* presents a new opportunity for further research as a potential treatment approach.

The results of this investigation showed that, compared to the Ct and T2 cell lines, the transmembrane protein *FLRT2* gene expression was higher in the A3 and A5 cell lines. This suggested that radiation alone and radiation in combination with estrogens could cause cancer throughout the malignant transformation process but not at the tumor stage. Such a result could corroborate the fact that *FLRT2* gene and protein expressions were higher in normal tissue than in tumor tissue. The authors demonstrated that *FLRT2* was implicated in breast cancer progression [33,34], specifically, the downregulation of *FLRT2* was associated with inactivated hypermethylation during tumor formation causing increased proliferation and cell migration in breast cancer. Due to this reason, it was considered a tumor-suppressor gene [34]. The overexpression of *FLRT2* had the opposite effect and decreased cell adhesion. It will remain a topic of study to determine how *FLRT2* expression behaves in patients with radiation-treated breast cancer. The databases indicated that, despite the aforementioned, patients with ER-positive breast cancer had greater levels of *FLRT2* gene expression, and it was positively correlated with *ESR2* gene expression in Basal and Luminal A breast cancer patients. These findings present a novel potential marker associated with estrogen receptor expression in breast cancer.

SLIT2 is a secreted polypeptide that guides the migration of cells expressing Roundabout 1 and 2 (ROBO1 and ROBO2) receptors. In the experimental model, *SLIT2* gene expression increased in response to radiation and dropped during the most severe stage (T2 cell line). This pattern correlated with the increased expression of genes and proteins in normal tissue compared to tumor tissue in the patient databases that were being studied. Regarding the effect of radiation on SLIT2 expression, it was reported that it was reduced after UVB irradiation in glioblastoma cell lines (U-373 MG and U-87 MG), determined by qPCR and immunoblotting [73,74]. However, the results found in this work are supported by other research indicating that the overexpression of *SLIT2* could inhibit breast tumor growth in vivo, suggesting its potential as a tumor-suppressor gene [75,76], activating the phagocytic activity of M1-like tumor-associated macrophages against breast tumor cells [77]. On the contrary, the loss of SLIT2 functions through gene deletion, epigenetic inactivation, and mutations could increase the risk of various cancers, including breast cancer [78,79]. Additionally, the overexpression of full-length *SLIT2* in MCF-7 cells inhibited breast tumor growth in nude mice, even in the presence of estrogen [75,76]. Despite the effect of estrogens on tumor growth in cells overexpressing SLIT2, the role of estrogen receptors in this process remains largely unknown. This work showed that *SLIT2* gene expression was higher in ER-positive breast cancer based on ER status. Furthermore, patients with Basal and Luminal A breast cancer had a positive correlation between the expression of the *ESR2* gene and *SLIT2*, presenting a new possible field to explore as a marker.

This study investigated the impact of radiation and estrogen on the expression of vanin-1, a cell surface hydrolase that has pantetheinase activity and produces amino-thiol cysteamine through the metabolism of pantothenic acid. Vanin-1 is important for metabolism, inflammation, and cellular stress [80]. *VNN1* gene expression was higher in the A3 and A5 cell lines than in the T2 cell line, corroborating the response to radiation before and during tumor formation but not in the malignant stage of the model. Vanin-1 is crucial in regulating the GSH-dependent response to oxidative injury from irradiation at the epithelial tissue level, suggesting a similar effect in the cells of our model [81]. The present findings indicated that the VNN1 gene and protein expressions were higher in normal tissue than in tumor tissue and had no correlation with *ESR1* gene expression; however, its gene expression was high in ER-negative patients. On the other hand, the *VNN1* and *ESR2* genes were positively correlated in Basal and Luminal A breast cancer patients. These findings are of great relevance since the role of VNN1 is largely unknown in breast cancer. In colorectal cancer, vanin-1 influences glutathione levels, impacting carcinogenesis. It is presumed that *VNN1* may have a similar effect on DNA damage and disrupt pathways involved in apoptosis, metabolism, and cell growth, such as Nrf2 and HIF-1α [82].

Another gene affected by radiation was *MYLK*, as determined by its increased expression in the A3 cell line compared to the Ct and T2 cell lines. MYLK is an element of the actin cytoskeleton and is involved in cellular processes, such as cell adhesions, migration, and survival [83]. The results showed that *MYLK* gene and protein expressions were higher in normal tissue than in tumor tissue. These results were consistent with a study by Kim and Helfman that demonstrated decreased MYLK expression in breast cancer and the absence of MYLK-disrupted cell–cell adhesion, which caused invasive behavior in breast epithelial cells [41]. Additionally, such a study associated *MYLK* expression with estrogen receptors. According to the findings, *MYLK* and estrogen receptor gene expression had a positive correlation with *ESR1* in patients with Her2 and with *ESR2* expression in patients with Luminal A breast cancer. However, there was no significant difference between ER-positive and -negative patients, according to the phenotypic ER status. Though the exact relationship between *MYLK* and estrogen receptors remains to be elucidated, understanding this relationship could be essential to comprehend how the motile phenotype of breast cancer cells is influenced by estrogen receptor expression.

Two proteins linked to the dynamics of the microtubule cytoskeleton, MAP1B and TUBA1A, were found to have unfavorable effects on patients with breast cancer when their expression levels were high in this study. The protein MAP1B binds, stabilizes, and controls microtubule dynamics. Histologically, the development of the nervous system was initially connected to MAPs, but since then, non-neural cancer tissues have been found to exhibit primary neuronal MAPs aberrantly [84]. The results indicated that A3 cells had higher *MAP1B* gene expression than the Ct and T2 cell lines, showing an early response of the cells to radiation. The *MAP1B* gene had a negative correlation with *ESR1* gene expression, and its expression was higher in ER-negative patients. This suggested that MAP1B expression and ERα were inversely correlated in patients with breast cancer. However, the *MAP1B* and *ESR2* gene expression levels were positively correlated in Luminal A breast cancer patients. Those patients also exhibited a significant reduction in the probability of survival when *MAP1B* expression levels were high. Such findings were supported by studies corroborating that *MAP1B* was highly expressed in the most aggressive and deadliest form of breast cancer, such as triple-negative breast cancer, but not in other subtypes. Even such expression was found to be highly correlated with poor prognosis [85]. Recently, Chien et al., 2020 showed that MAP1B overexpression was not only an indicator of unfavorable clinicopathological parameters but also an independent prognostic factor able to predict poor disease-specific survival and metastasis-free survival rates in patients with urothelial carcinoma of the upper tract and the bladder [86]. Given this background, new studies focused on relating the expression of MAP1B and ERβ in Luminal A breast cancer patients will be necessary to improve the understanding of these mechanisms.

On the other hand, the E and A3 cell lines had greater levels of TUBA1A gene expression than the A5 cell line. TUBA1A gene expression and ER status did not significantly correlate with estrogen receptor expression. Luminal B patients’ survival rates were found to be adversely correlated with its high expression. This aggressive phenotype overexpressing *TUBA1A* was reported in the paclitaxel-resistant MCF7 breast cancer cell line [87]. In another study, Tau, α-tubulin, and βIII-tubulin were analyzed by immunohistochemical expressions in 183 primary breast carcinoma patients who had undergone surgical resection, and the results indicated that the loss of α-tubulin expression was significantly associated with lymph node metastasis and distant metastasis [88].

The interruption in microtubule polymerization can lead to cryostasis and cell death by apoptosis. Thus, the interference with tubulin dynamics is a clinically proven approach for the development of highly efficient cancer chemotherapeutics [89,90,91,92]. The drugs that target tubulin and microtubules are among the most successful strategies in cancer chemotherapy [93,94]. Targets that aim to disrupt dynamic cellular processes have been successful in stopping the growth of malignancies; nevertheless, many of these therapeutic targets result in drug resistance. As demonstrated in this work, concentrating new targets on any stage of cell motility could be advantageous in preventing cancer progression.

The primary results, which are outlined in Table 2, show that every gene associated with motility reacted to radiation, sometimes in conjunction with estrogen.

This implies that the early initiation of a response to cell injury is contingent upon radiation. It might cause cellular movement that involves the remodeling and activation of cytoskeleton proteins, such as MAP1B, TUBA1A, and MYLK, resulting in the development of cellular protrusions that interact for force and adhesion with the extracellular matrix. Cell-surface proteases, such as ADAM12, VNN1, and FLRT2, would cleave ECM components with the aid of SLIT2, CYR61, and ADAM12 in response to mechanical stimuli, allowing for cancer cells to migrate more easily. This study also revealed key insights associated with genes and ER expression. Clinically, ERα expression is a crucial therapeutic marker for patients with breast cancer. Nevertheless, most of the genes examined here did not appear to be related to this receptor. However, the findings that associate ERβ (*ESR2* gene) with genes related to cell motility provide a fresh context for potential treatment strategies.

Additional studies and experiments will be needed to clarify how cell motility genes are connected to estrogen receptors and how ionizing radiation affects the expression of markers like the estrogen receptor during the malignant transformation of breast cancer. It has been demonstrated that exposure to ionizing radiation resulted in an increased risk of breast cancer, and there is strong evidence that steroid hormones influence radiosensitivity and breast cancer risk [95]. On the other hand, researchers have demonstrated that progesterone increases the quantity of proliferating cells with chromosomal damage and shields mammary cells from radiation-induced death [96].

Lastly, the survival of breast cancer patients was significantly influenced by the high expression of *TUBA1A* in Luminal B patients and *MAP1B* in Luminal A patients, which corroborated the importance of these markers and the expression of genes and proteins related to cell motility.

## 5. Conclusions

In this study, we used a breast cancer model to examine the effects of radiation alone or in combination with estrogens on the expression of genes linked to cell motility, such as *ADAM12*, *CYR61*, *FLRT2*, *SLIT2*, *VNN1*, *MYLK*, *MAP1B*, and *TUBA1A*. According to the suggested model, there was an early cellular response to radiation that was more significant in the process of initiation than tumor progression because the expression of these genes in the A3 cell line increased even more than that of the malignant T2 cell line. According to a bioinformatic study, the expression of the genes *FLRT2*, *SLIT2*, *VNN1*, *MAP1B*, *MYLK*, and *TUBA1A* was found to be higher in normal tissue than in malignant tissue in patients with breast cancer. However, the expression of *CYR61* and *ADAM12* was higher in tumors than in normal tissue, and they correlated negatively with the expression of the *ESR1* gene. Since the *ESR1* gene was only positively correlated with *MYLK* in Her2 patients, the relationship of these genes with *ESR2* gene expression was relevant. Concerning *ESR2* gene expression, a negative correlation with *CYR61* was observed, but it was positive with *FLRT2*, *MYLK*, *MAP1B*, and *VNN1*, offering a new background for possible therapeutic approaches. Finally, a decreased survival rate was observed in patients exhibiting high expression levels of *TUBA1A* and *MAP1B*. These genes also showed negative ER status, an important parameter for endocrine therapy. This work provides new targets focused on the cell motility process, and it also presents novel prospects based on hormonal receptor expression in breast cancer patients for exploring new therapeutic strategies.

## Figures and Tables

**Table 1:** Statistical significance of protein expression between normal sample and primary tumor.

Protein	Expression	*p*-Value
ADAM12	N > T	<1 × 10^−12^
CYR61	T > N	1.59799 × 10^−1^
FLRT2	N > T	1.11180 × 10^−4^
SLIT2	N > T	5.92435 × 10^−1^
VNN1	N > T	2.00554 × 10^−6^
MYLK	N > T	6.40369 × 10^−7^
MAP1B	N > T	5.79667 × 10^−12^

*p*-value was calculated by UALCAN portal, and its summary significance was expressed as a comparison between normal sample (N) and primary tumor (T).

**Table 2:** Summary of the main findings of this work.

Gene	Gene Expression	Tumor vs. Normal Tissue Gene Protein		*ESR1* and *ESR2* Correlation	ER Status	Survival
*ADAM12*	A3 > Ct/A5/T2	T > N	N > T	*ESR1*: (−) LumA/LumB *ESR2*: NS	(+)	NS
*CYR61*	A3 > Ct/T2	N > T	T > N	*ESR1*: (−) LumA/LumB *ESR2*: (−) Her2; (+) LumA	NS	NS
*FLRT2*	A3/A5 > Ct/T2	N > T	N > T	*ESR1*: NS *ESR2*: (+) Basal/LumA	(+)	NS
*SLIT2*	A3, A5 > Ct/T2	N > T	N > T	*ESR1*: NS *ESR2*: (+) Basal/LumA	(+)	NS
*VNN1*	A3, A5 > T2	N > T	N > T	*ESR1*: NS *ESR2*: (+) LumA/LumB	(−)	NS
*MYLK*	A3 > Ct/T2	N > T	N > T	*ESR1*: (+) Her2 *ESR2*: (+) LumA	NS	NS
*MAP1B*	A3 > Ct/T2	N > T	N > T	*ESR1*: NS *ESR2*: (+) LumA	(−)	(+) LumA
*TUBA1A*	E/A3 > Ct, A5	N > T	--	*ESR1*: NS *ESR2*: NS	(−)	(+) LumB

Ct: control, E: Estrogen, A3: Alpha3, A5: Alpha5, T2: Tumor2, T: Tumor, N: Normal, (−): Negative, (+): Positive, NS: non-significant.

## Data Availability

TIMER20 is freely available at http://timer.cistrome.org/ (accessed on 6 August 2023); UCSC Xena online exploration tools are freely available at https://xena.ucsc.edu/ (accessed on 20 August 2023). The data generated in the present study may be requested from the corresponding author.
